# Male‐biased dispersal in a fungus‐gardening ant symbiosis

**DOI:** 10.1002/ece3.7198

**Published:** 2021-01-28

**Authors:** Alix E. Matthews, Katrin Kellner, Jon N. Seal

**Affiliations:** ^1^ Department of Biology The University of Texas at Tyler Tyler TX USA; ^2^ Present address: College of Sciences and Mathematics and Molecular Biosciences Program Arkansas State University Jonesboro AR USA

**Keywords:** Attini, co‐dispersal, microsatellites, population structure, sex‐biased gene flow, symbiosis

## Abstract

For nearly all organisms, dispersal is a fundamental life‐history trait that can shape their ecology and evolution. Variation in dispersal capabilities within a species exists and can influence population genetic structure and ecological interactions. In fungus‐gardening (attine) ants, co‐dispersal of ants and mutualistic fungi is crucial to the success of this obligate symbiosis. Female‐biased dispersal (and gene flow) may be favored in attines because virgin queens carry the responsibility of dispersing the fungi, but a paucity of research has made this conclusion difficult. Here, we investigate dispersal of the fungus‐gardening ant *Trachymyrmex septentrionalis* using a combination of maternally (mitochondrial DNA) and biparentally inherited (microsatellites) markers. We found three distinct, spatially isolated mitochondrial DNA haplotypes; two were found in the Florida panhandle and the other in the Florida peninsula. In contrast, biparental markers illustrated significant gene flow across this region and minimal spatial structure. The differential patterns uncovered from mitochondrial DNA and microsatellite markers suggest that most long‐distance ant dispersal is male‐biased and that females (and concomitantly the fungus) have more limited dispersal capabilities. Consequently, the limited female dispersal is likely an important bottleneck for the fungal symbiont. This bottleneck could slow fungal genetic diversification, which has significant implications for both ant hosts and fungal symbionts regarding population genetics, species distributions, adaptive responses to environmental change, and coevolutionary patterns.

## Graphical abstract

A TCS haplotype network for *Trachymyrmex septentrionalis* constructed with COI DNA sequences. Populations of *T. septentrionalis* in Northern Florida are characterized by two haplotypes found in the panhandle and another found in the peninsula. These clusters are largely nonoverlapping since only two sequences clustering with the panhandle were found in the peninsula.

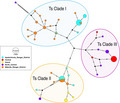

## 1 INTRODUCTION

Dispersal is a ubiquitous and fundamental life‐history trait that plays a role in the evolutionary ecology of organisms. Dispersal can shape population genetic differentiation, overall species distributions, and how species may respond or adapt to environmental change (Clobert et al., 2012; Ellis et al., 2015). For species with low dispersal capabilities, historical, ecological, or anthropogenic barriers to movement can influence their population genetic structure over certain scales (Doña et al., 2019; Haye et al., 2014; Manel et al., 2003; Storfer et al., 2010). For example, barriers may slow gene flow between some populations, increase reproductive and genetic isolation and ultimately result in genetically divergent populations (Clark et al., 2010; DiBlasi et al., 2018; Slatkin, 1987).

Within species, dispersal may be biased toward certain individuals, which has consequences for a species’ genetic diversity and population genetic structure. For example, in some species, female birds tend to disperse farther than males, while male mammals tend to disperse farther than females (sex‐biased dispersal) (Greenwood, 1980; Trochet et al., 2016). While females form the core of societies in ants (and all social Hymenoptera), considerable variation exists among the dispersal abilities of males and reproductive females (Bourke & Franks, 1995; Cronin et al., 2013; Hakala et al., 2019; Helms, 2018; Jacobs & Heinze, 2019; Keller et al., 2014). For example, some ant species have wingless individuals (females or males) that can move across scales of just a few meters, or winged individuals that vary in their ability or tendency to disperse long distances (over hundreds of meters). Typically, dispersal pattern and colony founding mode determine intraspecific population genetic patterns. For example, in species where queens do not fly, males may fly and be responsible for most long‐distance dispersal (Berghoff et al., 2008; Hakala et al., 2019). In other species, wingless males and winged females may experience inbreeding due to a lack of dispersal (Jacobs & Heinze, 2019). In species with winged males and females, both sexes may contribute to long‐distance dispersal, gene flow, and near panmictic population structure (Johansson et al., 2018). On the other hand, some species with volant sexuals nevertheless show signatures of male‐biased long‐distance dispersal (Holzer et al., 2009), especially in species with relatively large and fat‐laden queens that produce their first offspring via metabolic stores (claustral founding) (Helms, 2018).

The fungus‐gardening (attine) ants are common ants in low to mid latitudes of the western hemisphere (Branstetter et al., 2017; Nygaard et al., 2016; Seal & Tschinkel, 2006). The ants depend on their fungal cultivar as their main nutritional source, and the fungus depends on the ants for propagation, survival, and dispersal. Virgin queens (gynes) are largely responsible for dispersal of the mutualistic fungi (vertical transmission) by storing it in an infrabuccal pocket prior to the nuptial flight (Huber, 1905; Meirelles et al., 2016; Mueller, 2002; Mueller et al., 2001; Schultz & Brady, 2008). While horizontal movement of fungal symbionts among colonies may occur, it has not been extensively documented in the more derived lineages and it is unclear how this happens or how frequently it may occur (Ješovnik et al., 2017; Luiso et al., 2020; Mueller et al., 2018; Smith et al., 2019; Solomon et al., 2019). Moreover, horizontal movement of fungi likely occurs by ants moving over the ground (i.e., during raids (Adams et al., 2000)) and across the scales of meters as in dependent founding ant species that reproduce by budding (Hakala et al., 2019; Sanetra & Crozier, 2003). Thus, most long‐distance symbiotic fungal expansion and diversification is expected to be largely dependent on dispersal capabilities of the female ants. As a result, female‐biased dispersal (or at least not male‐biased dispersal) may be favored in attine ants; strict male‐biased dispersal would conflict with the dispersal interests of the fungus (Mueller, 2002) by limiting the expansion and diversification of the fungal symbiont. However, any conflicts could be resolved by males and gynes having similar dispersal abilities. Additionally, gynes of most attines appear to be energetically cheap to produce and most do not found claustrally (Fernández‐Marín et al., 2004; Seal, 2009; Seal & Tschinkel, 2007a); thus, attines might not be under selection to produce large, heavy gynes that are generally dispersal‐limited because of the energetic demands of flight (Helms, 2018). Consequently, the relatively lighter attine gynes may fly just as far as males, thus increasing expansion capabilities of the co‐dispersed fungus.

Surprisingly, there have been very few studies that examine sex‐biased dispersal and gene flow in fungus‐gardening ants, even though the molecular markers necessary to do so have been developed. For instance, microsatellite markers have been used to examine polyandry, polygyny, and population structure (Bekkevold et al., 1999; Fjerdingstad & Boomsma, 2000; Helmkampf et al., 2008; Kellner et al., 2013; Matthews et al., 2020; Murakami et al., 2000; Rabeling et al., 2011, 2013, 2014) and mitochondrial DNA (mtDNA) markers have been used to examine phylogeographic structure (i.e., female dispersal patterns) in several attine species (Cardoso et al., 2015; Seal et al., 2015; Solomon et al., 2008). While at least one study reported comparable dispersal abilities in both male and female *Atta colombica*, only a small portion of its known range was surveyed (Helmkampf et al., 2008). Indeed, *Atta* species are the only attines that exhibit claustral founding (Fernández‐Marín & Wcislo, 2005; Fernández‐Marín et al., 2004; Hölldobler & Wilson, 2011; Huber, 1905); it therefore remains possible that the large females (among the largest individual ants in the world) are not capable of flying as far as males.

In this study, we employ mtDNA markers and diploid microsatellite markers that were developed using whole genome sequencing (Matthews et al., 2020) to examine population structure, gene flow, and signatures of sex‐biased dispersal in a fungus‐gardening ant species, *Trachymyrmex septentrionalis* (McCook, 1881). *Trachymyrmex septentrionalis* is the only fungus‐gardening ant found entirely within the United States and is distributed from Texas to Florida to Long Island, New York, with a center of genetic diversity found in northern Florida (Matthews et al., 2020; Rabeling et al., 2007; Seal et al., 2015; Senula et al., 2019). Although northern Florida is near the southern edge of the distribution of *T. septentrionalis*, the population does not appear to exhibit traits of a peripheral population (Mueller et al., 2011; Sexton et al., 2009). Prior studies using mtDNA have shown significant phylogeographic structure across its range. For example, haplotypes are represented by phylogroups east and west of the Mississippi River, with western populations consisting of one clade whereas those found in the east are more diverse, consisting of at least three clades (Mikheyev et al., 2008; Seal et al., 2015). A preliminary analysis of nuclear microsatellites supports this overall pattern—higher diversity in populations east of the Mississippi River relative to the west (Matthews et al., 2020). *Trachymyrmex septentrionalis* is one of the most common and abundant ant species in the longleaf pine forests of Florida whose populations appear to respond rapidly to annual variations in climate (especially rainfall) and local conditions (e.g., relief) and often move significant amounts of soil in the process (Seal & Tschinkel, 2006, 2008, 2010; Tschinkel & Seal, 2016). Therefore, understanding the population genetics of this species will help us understand the dispersal biology of an ecologically important symbiosis.

## 2 MATERIALS AND METHODS

### 2.1 Study site and field methods

**FIGURE 1:**
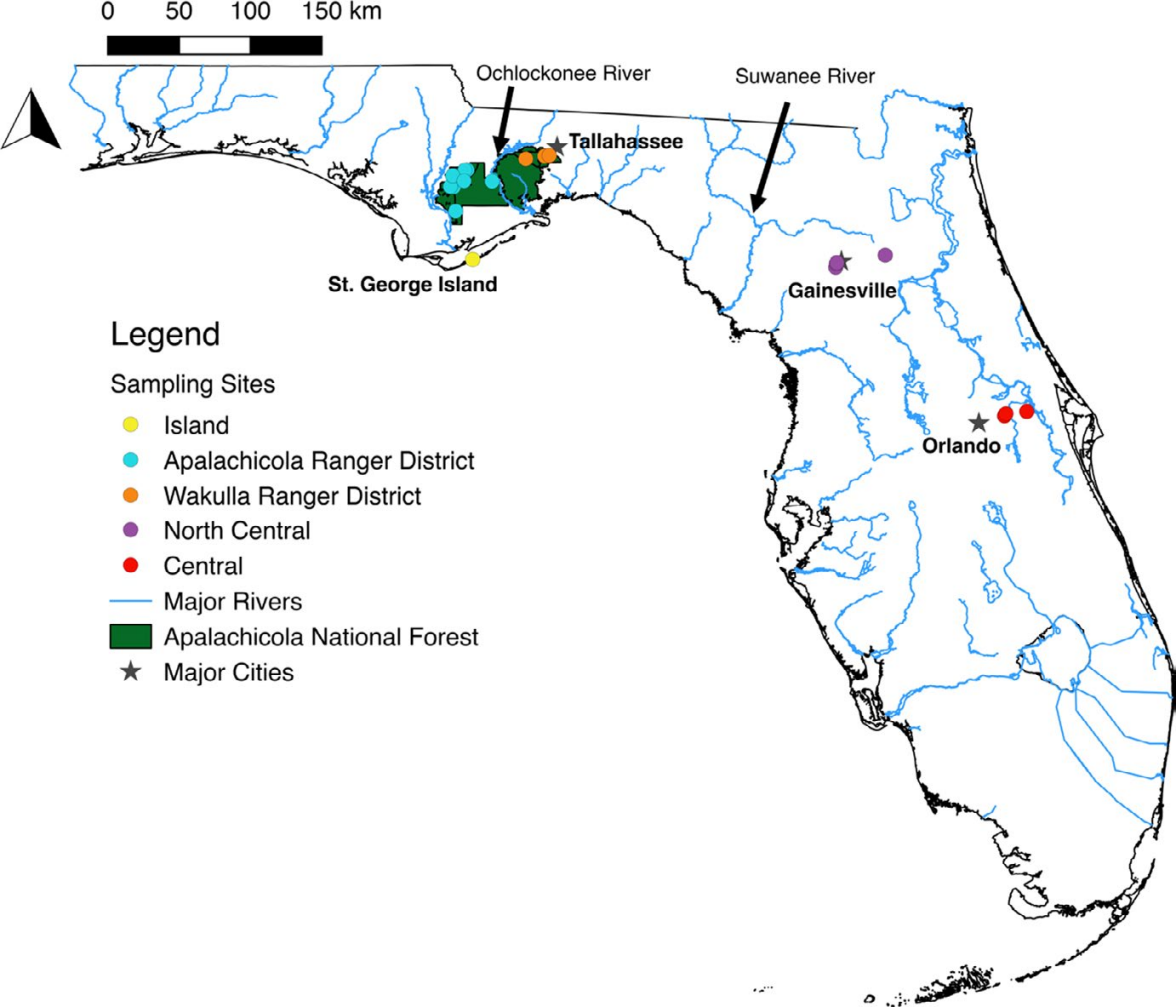
Localities of *Trachymyrmex septentrionalis* specimen sampling sites in this study, color‐coded by major region. The Apalachicola National Forest is highlighted in green. Major cities are denoted by gray stars and major rivers are labeled

*Trachymyrmex septentrionalis* colonies were found in Florida, USA. Florida is located entirely within the North American Coastal Plain, which is a global biodiversity hotspot owing to its unique geologic history and diverse ecosystems (Noss, 2012; Noss et al., 2015; Griffith et al., 1994). Samples were collected across two primary regions in Florida that correspond with known biogeographic breaks in the state (e.g., (Seal et al., 2015); the panhandle (which included two distinct ranger districts within the Apalachicola National Forest and a nearby barrier island) and the peninsula, represented by samples collected in North Central Florida near Gainesville and in Central Florida near Orlando (Figure 1).

In the panhandle, the Apalachicola National Forest (ANF) is divided into two ranger districts that occur on either side of the Ochlockonee River: the Apalachicola Ranger District on the west and the Wakulla Ranger District on the east. The Apalachicola Ranger District (ARD) is comprised mainly of pine flatwood forest with high herbaceous cover, little relief, and a high water table (Abrahamson & Harnett, 1990). *Trachymyrmex septentrionalis* colonies in pine flatwoods are restricted to “habitat islands” of somewhat higher elevation (relative to the water table) and population sizes likely vary temporally as annual rainfall varies from drought to very wet (Seal & Tschinkel, 2010). On the other hand, the Wakulla Ranger District (WRD) contains significant stands of sandhill forest (i.e., remnants of Pleistocene sand dunes; (Myers, 1990) that are excessively drained and support many species associated with deserts (Brown et al., 1990). *Trachymyrmex septentrionalis* colonies are found throughout the sandhills, absent only from frequently inundated areas near ephemeral ponds. Unlike flatwoods populations, those found in sandhills do not likely fluctuate very much in size, at least with respect to rainfall (Seal & Tschinkel, 2006, 2010). Both ranger districts are dominated and characterized by longleaf pine (*Pinus palustris*), xeric adapted oaks (e.g., turkey oak, *Quercus laevis*), and sandy soils. Located just south of the ANF, St. George Island is a sandy barrier island approximately 6km from the mainland. Despite its low relief and susceptibility to hurricanes, the island contains several stands of pine forest on elevated regions (Gornish & Miller, 2010; Huffman et al., 2004) which harbor several species typical of sandhills further inland, such as *T. septentrionalis* and the seed‐harvesting ant *Pogonomyrmex badius*. The Florida peninsula is separated from the panhandle by the Suwanee River, a known biogeographic barrier to many species (including ants), owing to different soils and geological history (Brown et al., 1990; Richter et al., 2014; Strehl & Gadau, 2004). Thus, even though *T. septentrionalis* colonies were sampled in similar upland habitats containing longleaf pine and turkey oaks, the collections were made across multiple ecological gradients and potential dispersal barriers.

*Trachymyrmex septentrionalis* colonies were identified in the field by their distinctive and steep crescent‐shaped mounds, which are built up by ants piling excavated chamber soil (Seal & Tschinkel, 2006). Individual worker ants were collected directly from colonies in 2013 and 2018. All workers were preserved in 95% ethanol at −20°C until DNA extraction. Only one individual worker per colony was used for sequencing. All relevant specimen information (e.g., collection date, region/location) can be found in Table S1.

### 2.2 Microsatellite genotyping and genetic diversity

We extracted genomic DNA for microsatellite genotyping from 64 individual *T. septentrionalis* workers using a QIAamp DNA Micro Kit (QIAGEN). We implemented the M13‐tail polymerase chain reaction (PCR) method (Schuelke, 2000), which involves three primers in the PCR: forward primers (Matthews et al., 2020) with an M13‐tail at the 5’ end, an unlabeled reverse primer, and a universal M13 primer labeled with 6‐FAM (6‐carboxy‐fluorescine) fluorescent dye. These were used to amplify nine microsatellite loci (Ts21, Ts25, Ts32, Ts33, Ts39, Ts41, Ts43, Ts46, Ts5) (Matthews et al., 2020).

PCRs were performed in a 10 μl mix containing 1 μl of 10X PCR buffer (1.0X; Applied Biosystems), 1 μl Bioline^®^ dNTP mix (1 mM each; 0.1 mM as a proportion of the total), 1.5 μl of 25 mM MgCl_2_ (3.75 mM; Applied Biosystems), 0.5 μl of 20 μM BSA (1 μM; New England Biolabs), 0.3 μl of 2 μM tag labeled primer (0.06 μM; forward primer with M13 tag), 0.6 μl of a 2 μM universal dye‐labeled primer (0.12 μM; FAM label with M13 tag), 1 μl of 2 μM unlabeled primer (0.2 μM; reverse primer), 0.1 μl of *Taq* polymerase (0.5 U; Applied Biosystems), and 1 μl of DNA template. Nuclease‐free water was used to make up the remaining volume. The following thermocycling profile was used on an Eppendorf Mastercycler: initial denaturation of 4 min at 95°C, followed by 25 (or 30) cycles of 30 s at 95°C, 45 s at the primer‐specific annealing temperature found by a temperature gradient program, 45 s at 72°C, then eight cycles of 30 s at 95°C, 45 s at 53°C, and 45 s at 72°C, followed by a final extension of 5 min at 72°C. Diluted PCR products were run on an Applied Biosystems 3730 Genetic Analyzer and fragments were sized with LIZ600 size standard at the University of Texas at Austin DNA Sequencing Facility in Austin, Texas, USA. We scored alleles using Geneious v10.2.3 (Kearse et al., 2012).

For each locus, we used GenAlEx v6.5 (Peakall & Smouse, 2006, 2012) to estimate the number of alleles (*K*), observed and expected heterozygosity (H_o_ and H_e_), and the probability of identity (PI; the probability of two independent samples having the same genotype). We assessed deviations from Hardy–Weinberg equilibrium (HWE) expectations using GENEPOP v4.2 (Rousset, 2008) across each locus for the overall dataset, as well as across each locus within each population. GENEPOP was also used to test for linkage disequilibrium (LD) across all pairs of loci.

### 2.3 Amplification and sequencing of mtDNA

In order to augment our microsatellite data, we included previously sequenced mtDNA data from Seal et al. (2015; *n* = 61) and sequenced an additional 26 samples directly for this study (total *n* = 87). A total of 55 samples had both microsatellite and mtDNA sequenced (Table S1). Again, DNA was extracted from whole individual workers using a QIAamp DNA Micro Kit (QIAGEN), and a 779‐bp sequence was obtained from the COI‐tRNA Leucine‐COII region of mtDNA. We used the following primers: C1‐J2195 (alias CO1‐RLR; 5′‐TTGATTTTTTGGTCATCCAGAAGT‐3′); and C2‐N‐3661 (alias Barbara; 5′‐ CCACAAATTTCTGAACATTGACCA‐3′ (Simon et al., 1994). PCR mixtures and cycling profiles were identical to Seal et al. (2015). PCR products were then purified and sequenced at the University of Texas at Austin's DNA Sequencing Facility on an Applied Biosystems 3730 DNA Analyzer. Chromatograms were visually checked and resolved in Geneious v10.2.3 (Kearse et al., 2012), and sequences were aligned using MEGA v6.06 (Tamura et al., 2013) using the ClustalW algorithm (Thompson et al., 1994). New sequences are deposited into GenBank under accession numbers MN088095–MN088120 (Table S1). mtDNA is known to have several properties that make evolutionary conclusions problematic, such as pseudogenes or nuclear insertions (numts) (Beckenbach, 2009; Cristiano et al., 2014; Martins et al., 2007; Toews & Brelsford, 2012). Therefore, we examined for stop codons in our sequences (other than at the COI‐tRNA Leucine transitions) (Seal et al., 2015). Sequences were also long and frequently readable at >800 bp. Two primary advantages of mtDNA sequences are that (a) sequences can be readily obtained and (b) the problems associated with mtDNA are understood and can be easily examined, whereas the problems associated with nuclear markers are more uncertain (Bowen et al., 2014; Moreau, 2009). Moreover, a preliminary examination of the microsatellite markers of *T. septentrionalis* used in this study supported one of the main findings of mtDNA‐based studies in this species: pronounced genetic differentiation across the Mississippi River Valley (Matthews et al., 2020), thus making it unlikely that the COI sequences analyzed were numts, which usually lack variation because of purifying selection in nuclear genomes (Martins et al., 2007). Therefore, mtDNA sequences in this species are likely robust genetic tools (Matthews et al., 2020; Mikheyev et al., 2008; Seal et al., 2015).

### 2.4 Population genetic analyses

#### 2.4.1 Microsatellites

We measured population genetic structure by analysis of molecular variance (AMOVA) (Excoffier et al., 1992) and by pairwise Fst values between populations in Arlequin v3.5.2.2 (Excoffier & Lischer, 2010). Significance was tested using 1,000 permutations. We generated several subsets to examine signatures of population differentiation related to the varying ecologies and potential barriers to dispersal across our collection sites. Specifically, our subsets were as follows: (a) all regions; (b) all regions with the island excluded; (c) ANF panhandle regions only (i.e., ARD and WRD); (d) peninsular regions only (i.e., North Central near Gainesville and Central near Orlando); and (e) ANF regions as one collective region and peninsular regions as one collective region. For each subset, we adjusted the allowed missing data (from 0.15 to 0.5) in order to include all nine loci in all analyses.

To further assess population genetic structure, we used the Bayesian clustering algorithm implemented through STRUCTURE v2.3.4 (Pritchard et al., 2000), which uses allele frequency data to assign individuals into genetic clusters (*K*). These analyses were executed with no prior population information and under the admixture model with correlated allele frequencies. In order to determine *K*, we assessed *K* = 1 through *K* = 10 with default parameters. To explore each *K* value, we performed 10 replicates with a burn‐in value of 50,000 and 500,000 MCMC iterations. We determined the optimal *K* value by assessing the maximum likelihood values and Δ*K* (Evanno et al., 2005) implemented in Structure Harvester v0.6.94 (Earl & von Holdt, 2012). We then averaged the 10 STRUCTURE replicates for the optimal *K* through the CLUMPAK pipeline (Kopelman et al., 2015) and visualized the results in DISTRUCT v1.1 (Rosenberg, 2004).

We performed Mantel tests to test whether genetic distances correlate with geographic distance (isolation by distance; IBD). We generated several subsets to test for IBD. Specifically, we considered the following: (a) all sampling sites across all regions; (b) all sampling sites excluding the island; (c) all sampling sites within the ANF; and (d) all sampling sites within the peninsula. For measures of genetic distances, we used pairwise Fst values obtained from Arlequin and linearized them as Fst/(1 − Fst) (Rousset, 1997). Negative Fst values, if present, were set to zero. Otherwise, the absolute difference between values would be inflated, when in actuality they are effectively zero (i.e., no genetic differentiation). We used Geographic Distance Matrix Generator v1.2.3 (Ersts, 2013) to generate pairwise geographic distances (in km) from the GPS coordinates of the sampling sites. We implemented Mantel tests using the ade4 package (Dray & Dufour, 2007) in R (Team, 2018) with 9,999 permutations.

#### 2.4.2 Mitochondrial DNA

Using the COI mtDNA alignment, we examined haplotype diversity and calculated basic genetic polymorphism statistics and across regions and within regions using DNAsp v6 (Rozas et al., 2017). We then reconstructed a haplotype network for the COI data (*n* = 87; combined samples from Seal et al. (2015) and new sequences directly from this study) using the TCS method (Clement et al., 2000) in PopArt v1.7 (Leigh & Bryant, 2015).

## 3 RESULTS

### 3.1 Genetic diversity of microsatellite DNA

The mean number of alleles per locus across nine loci in regions of Florida was 18.9. The mean observed heterozygosity was 0.688, while the mean expected heterozygosity was higher at a value of 0.859 (Table 1). After Bonferroni correction for multiple comparisons across all regions, four of the nine loci showed deviations from expectations under HWE (Table 1). However, by region and locus, only four HWE tests were significant after Bonferroni corrections and no loci deviated in more than two regions (Table 1). Therefore, we retained all nine loci for downstream analyses. There were no cases of linkage disequilibrium detected for any pair of loci.

**TABLE 1:** Details of the nine polymorphic microsatellite loci analyzed for *Trachymyrmex septentrionalis* ants across Florida

Locus	All regions					SGI		ARD		WRD		North Central		Central	
	N	K	Ho_L_	He_L_	PI_L_	Ho	HWE	Ho	HWE	Ho	HWE	Ho	HWE	Ho	HWE
Ts5^a^	53	12	0.585	0.827	0.051	0.750	0.771	0.654	0.006	0.538	0.005	0.500	1	0.250	0.142
Ts21^a^	50	11	0.66	0.823	0.048	0.000	0.009	0.852	0.014	0.700	0.409	0.167	0.011	0.667	0.599
Ts25	61	22	0.787	0.829	0.028	0.667	1	0.778	0.039	0.769	0.586	0.818	0.163	0.857	0.571
Ts32	61	16	0.754	0.864	0.031	0.500	0.428	0.769	0.199	0.846	0.401	0.636	0.014	0.857	0.297
Ts33	63	14	0.714	0.776	0.072	0.400	0.300	0.778	0.110	0.692	0.929	0.909	0.089	0.429	0.174
Ts39^a^	63	22	0.492	0.919	0.012	0.667	0.413	0.519	***0***	0.583	0.002	0.273	***0***	0.429	0.012
Ts41	60	17	0.8	0.843	0.037	0.500	0.214	0.792	0.364	0.833	0.116	0.909	0.553	0.857	0.556
Ts43^a^	55	34	0.6	0.937	0.007	1.000	1	0.609	***0***	0.500	***0***	0.636	0.008	0.333	0.003
Ts46	59	22	0.797	0.921	0.011	0.500	0.003	0.833	0.119	0.833	0.180	0.800	0.478	0.857	0.052

Note

Superscript ^a^ indicates deviation from Hardy–Weinberg expectations after Bonferroni corrections. Bolded and underlined *p*‐values indicate deviation after Bonferroni correction.

Abbreviations: ARD, Apalachicola Ranger District; H_eL_, expected heterozygosity per locus for all regions; H_o_, observed heterozygosity per region by locus; H_oL_, observed heterozygosity per locus for all regions; HWE, Hardy–Weinberg Equilibrium *p*‐values per region by locus; K, number of alleles observed; *N*, the number of individuals genotyped; PI_L_, probability of identity per locus for all regions; SGI, St. George Island; WRD, Wakulla Ranger District.

### 3.2 Population genetic analyses

#### 3.2.1 Microsatellites

Overall, AMOVAs did not reveal significant population differentiation (overall Fst values were between 0.000 and 0.035 and all associated Fst *p*‐values were > 0.05). Thus, the amount of genetic variation attributable to differences between the assigned population subsets was low and insignificant; most of the genetic differentiation occurred within and among individuals (Table S2).

**FIGURE 2:**
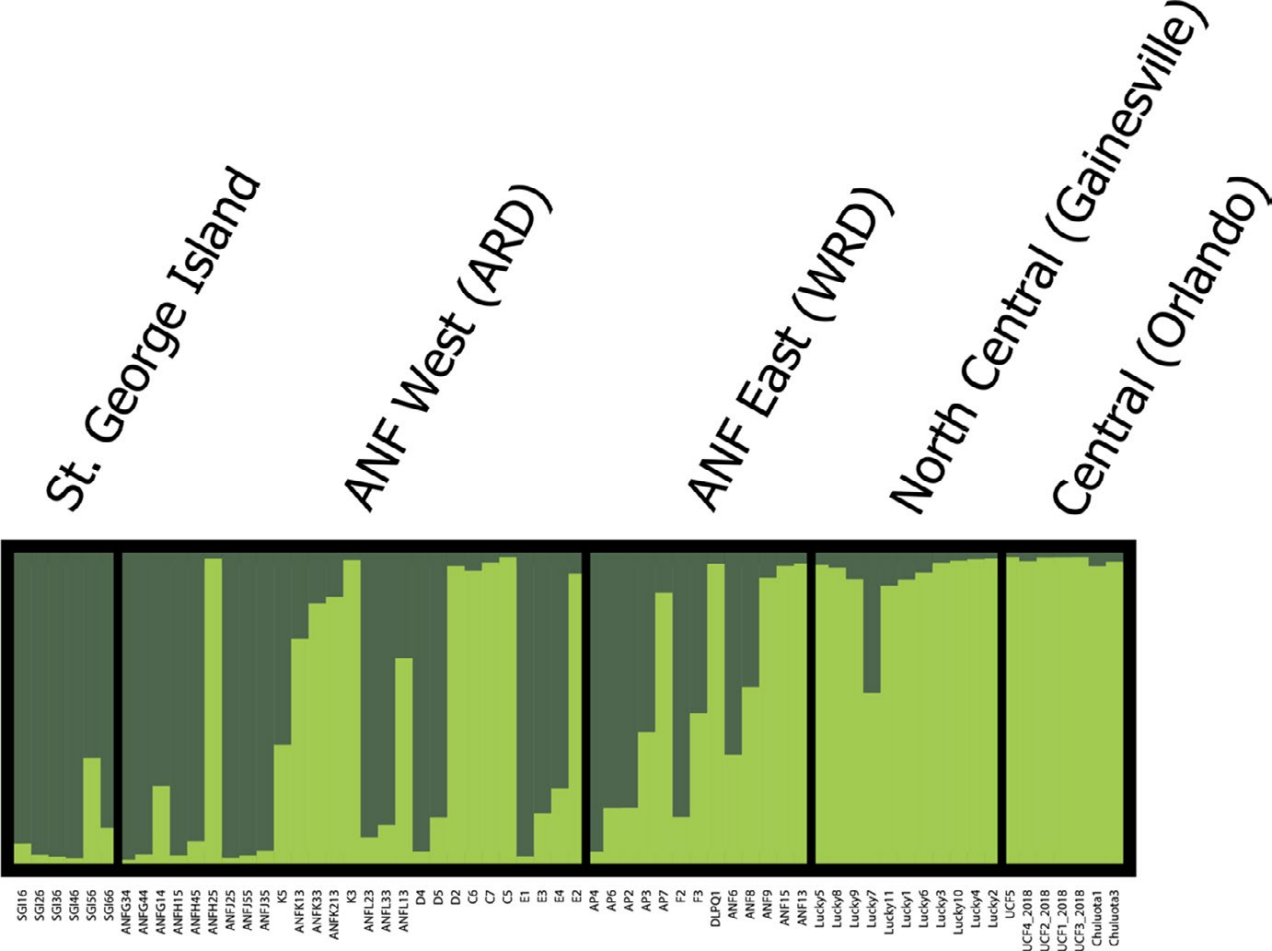
Bayesian model‐based genetic clusters (*K* = 2) inferred from STRUCTURE analyses using nine microsatellite loci from 64 *Trachymyrmex septentrionalis* ants across Florida. Each vertical bar represents an individual and each section between black bars represents major regions, which are labeled above. The two colors represent the two unique genetic clusters, and the proportion of each color represents the assignment probability of that individual to each cluster

Alternatively, according to our STRUCTURE results, there are two distinct genetic clusters (*K* = 2) in our dataset, providing evidence for genetic differentiation across Florida with signatures of gene flow between clusters. Specifically, the samples from St. George Island were largely assigned to one cluster. Likewise, the samples from the peninsula region were largely assigned to another single cluster with minimal admixture. The samples from the ANF appear to have substantial (nearly 50% overall) admixture of alleles associated with the peninsula and St. George Island clusters (Figure 2).

**FIGURE 3:**
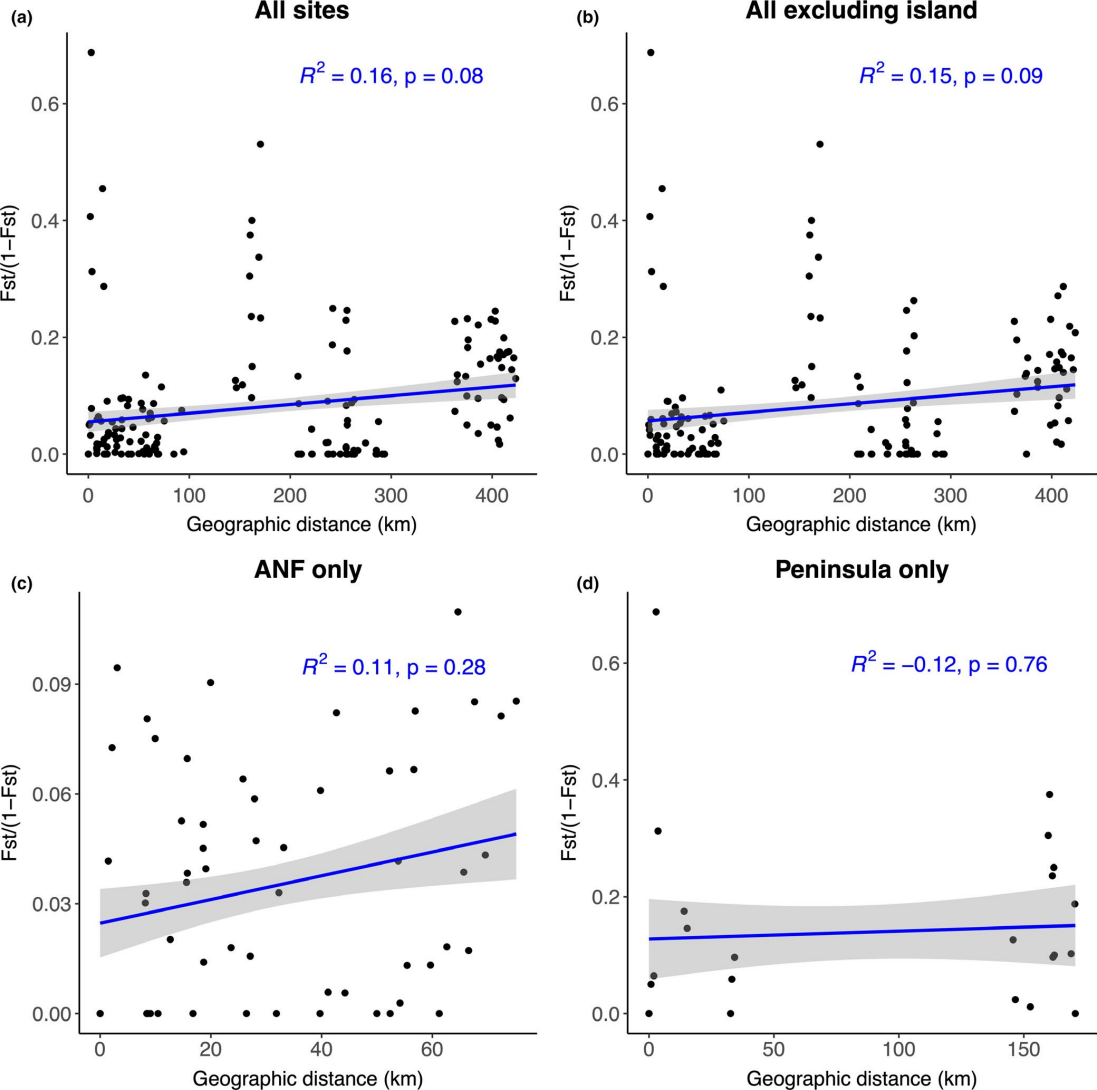
Isolation by distance scatterplots showing the relationship between genetic differentiation (transformed pairwise Fst values) and geographic distance (distance between sites in km) as identified by Mantel tests for *Trachymyrmex septentrionalis* ants in Florida. The analyses were conducted across (a) all sampling sites across all regions, (b) all sampling sites excluding SGI, (c) all sampling sites within the ANF only, and (d) all sampling sites within the peninsula only. Regression coefficients and *p*‐values are displayed for each analysis

Finally, based on results of the Mantel tests of the association between genetic distance and geographic distance, we found little evidence of IBD across the different subsets (Figure 3). Since the lowest *p*‐value was 0.08, it is risky to conclude no effect because of the risk of a Type II error when *p*‐values are between 0.05 and 0.20 (Underwood, 1997). Thus, the amount of genetic variation explained by geographic distance is low, suggesting that the genetic differentiation of our samples is continuous across geography rather than illustrating discrete populations separated by specific barriers.

#### 3.2.2 Mitochondrial DNA

**FIGURE 4:**
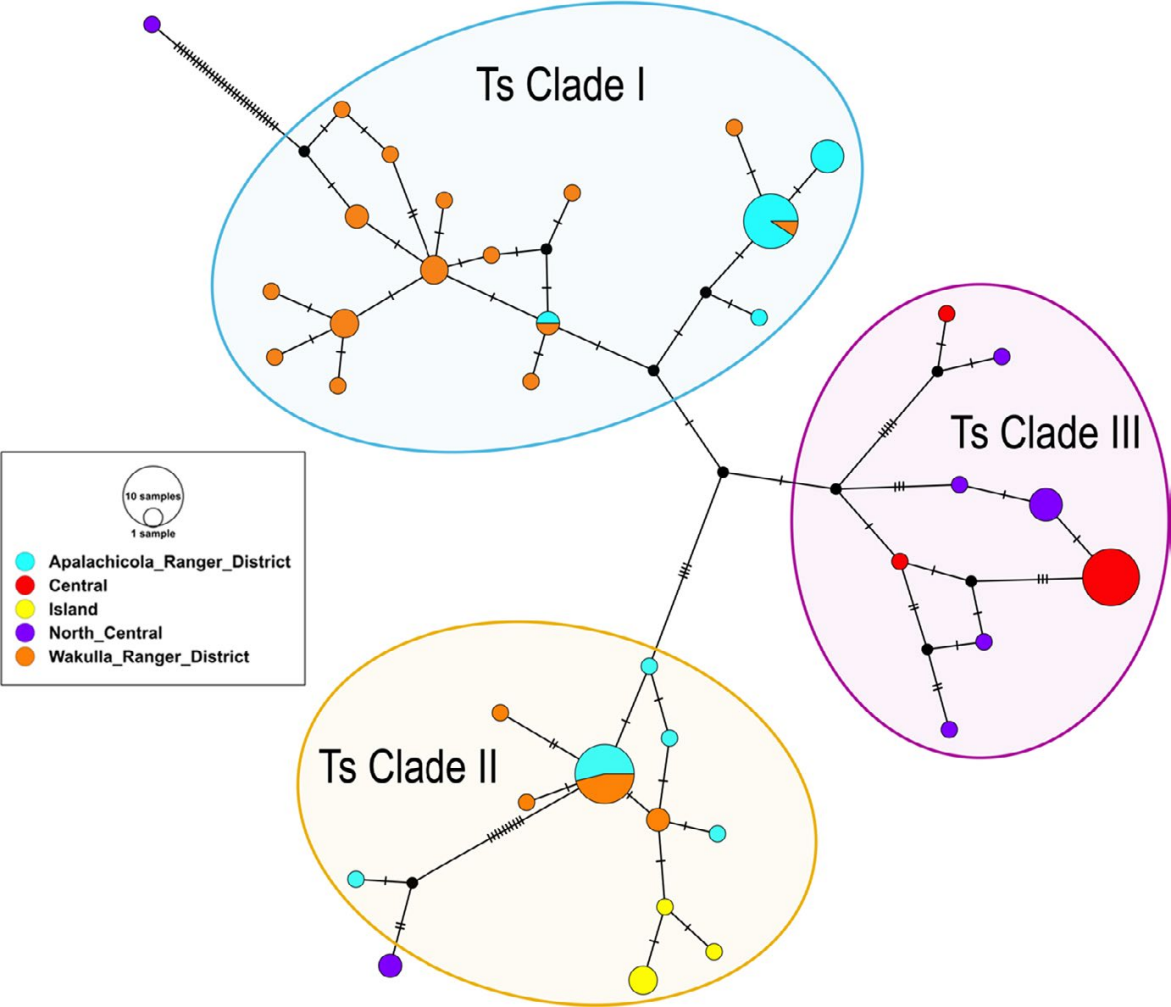
A TCS haplotype network for *Trachymyrmex septentrionalis* constructed with COI mitochondrial DNA sequences. Each circle represents a unique haplotype and the relative size of circles is proportional to the number of individuals harboring that haplotype. Colors correspond to sampling sites and align with Figure 1. Tick marks on branches indicate the number of mutations between haplotypes. Small black circles represent predicted intermediate haplotypes. Haplotype clades (Ts Clades I–III) are circled, highlighted, and labeled

In contrast to the diploid microsatellite markers, mtDNA markers indicated pronounced population differentiation. There were two distinct haplotype clades found in the panhandle and one in the peninsula. Three specimens collected in the North Central peninsula (Gainesville) appeared to be most similar to those found in the panhandle, however, even these were genetically unique (Figure 4). Across the combined 87 samples, there were 38 mitochondrial haplotypes identified and were defined by 71 polymorphic sites, which consisted of 33 singleton variable sites and 38 parsimony informative sites. Overall haplotype diversity was high and overall nucleotide diversity was low, with the same being true within each region (the Central region is an exception to high haplotype diversity), suggesting a recent population expansion (Table 2).

**TABLE 2:** Population genetic diversity indices of *Trachymyrmex septentrionalis* ants across Florida, as well as separated by site with major region indicated in parentheses

Region	*n*	*h*	hd (*SD*)	*π* (*SD*)	*k*
All	87	38	0.940 (0.013)	0.010 (0.00097)	8.1
Island (Panhandle)	5	3	0.700 (0.218)	0.001 (0.00045)	1
Apalachicola Ranger District (Panhandle)	27	9	0.795 (0.054)	0.007 (0.00093)	5.3
Wakulla Ranger District (Panhandle)	30	19	0.947 (0.026)	0.007 (0.00052)	5.7
North Central (Peninsula)	11	7	0.873 (0.089)	0.018 (0.00583)	14.2
Central (Peninsula)	14	3	0.275 (0.148)	0.002 (0.00137)	1.8

Abbreviations: *h*, number of unique haplotypes; hd, haplotype diversity; *π*, nucleotide diversity; *k*, the average number of nucleotide differences; *n*, sample size; *SD*, standard deviation.

## 4 DISCUSSION

The goals of this study were to examine the population structure, gene flow, and signatures of sex‐biased dispersal in a fungus‐gardening ant species across several ecological gradients in Florida, USA. Overall, our results suggest that most long‐distance dispersal is conducted by males. Specifically, females appear to have more limited dispersal as the mtDNA haplotypes are strongly geographically clustered (Figure 4). There were two COI clades found primarily in the Florida panhandle (Ts Clade I and Ts Clade II) and one that was restricted to the Florida peninsula (Ts Clade III). There were only three ants collected on the peninsula that somewhat clustered with panhandle ants: one with Ts Clade I (however, this specimen was separated from the Ts Clade I by 32 mutation steps) and two with Ts Clade II, separated by 12 mutation steps. These three ants were notably collected in North Central Florida, near Gainesville, geographically closer to the panhandle than the samples collected in Central Florida near Orlando. In contrast to mtDNA data, biparental microsatellite markers suggest significant gene flow across Florida (approximately 360km, linear distance between the Central region in Orlando and St. George Island, Florida) and minimal spatial structure (Figures 2 and 3). These results indicate considerable admixture of microsatellite alleles across the range of this study, which considering the geographic clustering of mtDNA COI sequences, likely arise from male movement. Together, these results suggest that males are responsible for most long‐distance dispersal while females (and concomitantly, their co‐dispersed fungal symbionts; (Tesson et al., 2015) do not disperse very far. This result was surprising since *T. septentrionalis* queens are not relatively large nor especially endowed with fat stores, which could impact their flying ability (Helms, 2018; Seal, 2009; Seal & Tschinkel, 2007a).

It is not clear how much of a barrier that rivers present to females since we did find evidence of some trans‐river female dispersal; however, the distance that females seem to move across rivers is much shorter relative to the distance males appear to disperse. The Suwannee River may be an important barrier to female dispersal, though not impenetrable since two peninsular haplotypes (in three individuals) were found clustered with the two panhandle clades (Figure 4). Conversely, the Ochlocknee River in the panhandle may not be a significant dispersal barrier to either sex considering the extensive admixture of microsatellite alleles (Figure 2) and shared haplotype clades (Ts Clades I and II; and even identical haplotypes in some cases) in both the ARD and WRD (Figure 4). The latter finding is surprising considering the differing ecologies and environments (i.e., frequently flooded flatwoods in the ARD and dry, xeric sandhills in the WRD). That being said, the finding of reduced genetic diversity in the ARD relative to the WRD (Table 2) could suggest recent expansion in the WRD. As a result, on small scales (10s of kilometers), *T. septentrionalis* appears to be a very mobile species, capable of rapid population growth and extensive dispersal capabilities, but there are limits to their expansion abilities across larger scales (>100s of kilometers). Possible explanations for this conclusion could be related to Pleistocene bottlenecks and then subsequent expansion, and a subsequent time lag in the expansion of COI haplotypes. Therefore, it would appear likely that males have a greater dispersal capability than females. Field studies measuring the variation in flight distance within and between sexes could further inform our results.

Evidence is currently lacking as to whether male‐biased dispersal is the general rule in the tribe Attini. This is surprising considering how important female dispersal is for the range expansion and ultimately evolution of the fungal symbiont (Mueller et al., 2001). For example, the basal neoattine *Mycetophylax simplex* exhibited relatively minor mtDNA (COI) variation across its range in Brazilian Atlantic Forest (Cardoso et al., 2015), which suggests that females are capable of long‐distance dispersal. However, as a lower attine (i.e., an early branching lineage of attini), *Mycetophylax* likely has smaller queens than *Trachymyrmex* sensu lato ants and other members of the so‐called “higher attini” (Seal, 2009); thus, the energetic cost of dispersal for *Mycetophylax* compared to *Trachymyrmex* sensu lato could possibly be lower. As another example, *Mycocepurus smithii* indicated stronger gene flow and little spatial structure in populations across the Panamanian isthmus (inferred from microsatellites) unlike their fungal symbionts that were more spatially structured, though the study did not also employ mtDNA markers like the present study (Kellner et al., 2013). While spatially structured fungal symbionts could point to limited female dispersal and long‐distance male dispersal like we found with *T. septentrionalis*, males are rare if not absent in *M. smithii*, which exhibit thelytokous parthenogenesis in Panama (Kellner et al., 2013). Thus, *M. smithii* female movement (and some level of disruption to vertical fungi transmission) likely explains the patterns in central Panama. While lower attines such as *Mycetophylax* and *Mycocepurus* cultivate fungi that are likely capable of independent life, fungi grown by higher attini such as *Trachymyrmex* and *Atta* are not (Schultz & Brady, 2008). Solomon et al. (2008) reported mtDNA (COI) clusters in three *Atta* species across continental scales, which suggests limited female dispersal, but did not examine whether males were capable of dispersing longer distances. Interestingly, ddRADseq (i.e., diploid markers) in *Atta texana* showed evidence of spatial structure and isolation by distance across a north‐south gradient in Texas (850 km). Though fungal symbionts also illustrate significant north‐south differentiation in this species, the patterns are not concordant with their host ants (Mueller et al., 2011; Smith et al., 2019), which could indicate independent/differential dispersal patterns of males, females, and fungal symbionts via unknown mechanisms.

Our results support greater dispersal abilities in male *T. septentrionalis* than females. Consequently, this suggests that the dispersal abilities of the vertically transmitted symbiotic fungus (and further associated microbial symbionts; (Ishak et al., 2011; Ronque et al., 2020) are likely also limited and thus also exhibits spatial structure, unless the fungus also has the ability of independent dispersal as suggested in *A. texana* (Smith et al., 2019). While higher attine fungi are not known to be free living (Nygaard et al., 2016), horizontal movement of fungi could occur among neighboring colonies as documented in less derived attini, that is, by garden sharing or stealing following losses due to predation or pathogen attack (Adams et al., 2000; Green et al., 2002; Kellner et al., 2013). As a result, fungi are likely to be even more dispersal limited by dependence on females who fly on the scale of a few kilometers or by sharing among neighboring colonies on the scale of meters. Ultimately, this could result in fungal populations that are more locally adapted to environmental conditions than the ants since male ants are contributing to longer distance gene flow (hundreds of kilometers) and can thus offset limited dispersal of females. While some of the variation in this study could be evidence of local adaptation, the environments from which we sampled are somewhat similar in terms of latitude, soils, and host plants (Myers, 1990), relative to the entire distribution and range of conditions that could be experienced by *T. septentrionalis* (Rabeling et al., 2007; Senula et al., 2019).

Limited female and symbiont co‐dispersal could represent a significant bottleneck to fungal diversification (and associated microbes). Bottlenecks are a common feature among vertically transmitted symbionts, which generally exhibit eroded genetic variation and reduced genomes compared to horizontally exchanged relatives (Bennett et al., 2014; Douglas, 2010; Helms et al., 2019; Nikoh et al., 2011). Bottlenecks may not only influence population demographics but also the adaptive abilities of co‐dispersed symbionts under varying environments. Consequently, the overall coevolutionary patterns and associations observed in the fungus‐farming ant symbiosis may be constrained by limited female dispersal especially in the higher attini that are characterized by obligate symbionts and large‐bodied, fatter queens.

The approximately 49 ant species in the genus formerly known as *Trachymyrmex* (now split into three genera; (Solomon et al., 2019) grow conservatively 4–5 phylotypes of fungi (Ješovnik et al., 2017; Luiso et al., 2020; Solomon et al., 2019). One possible explanation is that ant host diversification in these derived lineages has happened at a faster rate than their fungal symbionts because of limited female ant dispersal. Whether attine ants and their fungal symbionts have different evolutionary (or expansion) rates is currently unknown. The most recent genome‐level examinations suggested that attine fungal genomes have lower diversity of metabolic genes compared to free‐living fungi; however, this was based on transcriptomes (measures of gene expression) as we lack fully annotated attine fungal genomes because attine fungi are functionally polyploid (Kooij et al., 2015; Kooij et al., 2015; Nygaard et al., 2016). Alternatively, since neither ant nor fungi have to evolve at similar rates, reduced fungal lineage diversity could be due to higher evolutionary and subsequent extinction rates among the fungi, such that fungal diversification may occur more rapidly with ants adopting novel fungal strains and discarding others as climate and parasite pressure change the outcome of the interaction (Mehdiabadi et al., 2006; Seal & Mueller, 2014; Seal et al., 2014; Seal & Tschinkel, 2007b). Furthermore, some phylogenetic analyses suggest that the fungal lineages typically grown by leaf‐cutter ants (i.e., Clade A fungi grown by *Atta* and *Acromyrmex*) (Mueller et al., 2018) are younger than the ant lineages (Mikheyev et al., 2010; Nygaard et al., 2016) which suggests a recent domestication event. However, analyses of more recent datasets have called this conclusion into question since some non‐leaf‐cutting ants grow Clade A fungi (Mueller et al., 2017, 2018; Schultz et al., 2015), indicating that the fungi may have been around for as long as the less derived *Trachymyrmex* ants.

## 5 CONCLUSION

The differential dispersal patterns observed here between male and female *T. septentrionalis*, uncovered through an integration of microsatellite and mitochondrial DNA markers, indicate that males are responsible for most long‐distance dispersal in this species, while females are more dispersal limited. Specifically, matrilineal mitochondrial DNA illustrated spatially clustered haplotypes (i.e., no evidence of female long‐distance dispersal), while microsatellite data illustrated gene flow and minimal spatial structure across the study region (i.e., support for male‐biased dispersal). Consequently, these results have implications for expansion and diversification of the obligate fungal symbiont, which is largely dependent on the dispersal capabilities of female ants. A genetic bottleneck or constrained diversification of fungi may have significant implications for the coevolutionary patterns in this mutualistic symbiosis overall. A complementary analysis of fungal population structure, gene flow, and potential local adaptation would allow for this possibility to be explored in more detail. This study encourages further genetic, field, and experimental examinations of the dispersal biology of this species and other ecologically important fungus‐gardening ants, which will vastly improve our ability to understand and ultimately predict how host and symbiont populations expand and evolve across larger geographic and macroevolutionary scales.

## CONFLICT OF INTEREST

The authors declare no conflicts of interest.

## AUTHOR CONTRIBUTIONS

**Alix E. Matthews:** Conceptualization (equal); data curation (lead); formal analysis (lead); investigation (equal); writing – original draft (equal); writing – review and editing (equal). **Katrin Kellner:** Conceptualization (equal); formal analysis (supporting); funding acquisition (supporting); writing – review and editing (supporting). **Jon N. Seal:** Conceptualization (lead); formal analysis (supporting); investigation (lead); methodology (equal); project administration (lead); writing – original draft (lead); writing – review and editing (lead).

## Supporting information

Table S1

Table S2

## Data Availability

COI sequence data corresponding to the samples used in this study are deposited in GenBank under accession numbers MN088095‐MN088120, KP282952‐KP282961, and KP282964‐KP283014 and can be found in Table S1. Microsatellite genotypes for all individuals are archived and available in Dryad (https://doi.org/10.5061/dryad.fj6q573t2).
